# Supplementary material for: Ongoing evolution of the Mycobacterium tuberculosis lactate dehydrogenase reveals the pleiotropic effects of bacterial adaption to host pressure
Source: PLoS Pathog. 2024 Feb 29;20(2):e1012050. doi: 10.1371/journal.ppat.1012050 (PMC10931510; doi:10.1371/journal.ppat.1012050)
Supplement: S1 Fig — Each dot denotes the average of three technical replicates, the bars represent the standard deviation. The p-value indicates the results of ordinary-one way ANOVA tests conducted at each time point for each media condition, comparing the OD600 of the ancestral and variant strains. Sidak’s multiple comparison correction was performed. (PDF) [file ppat.1012050.s001.pdf]

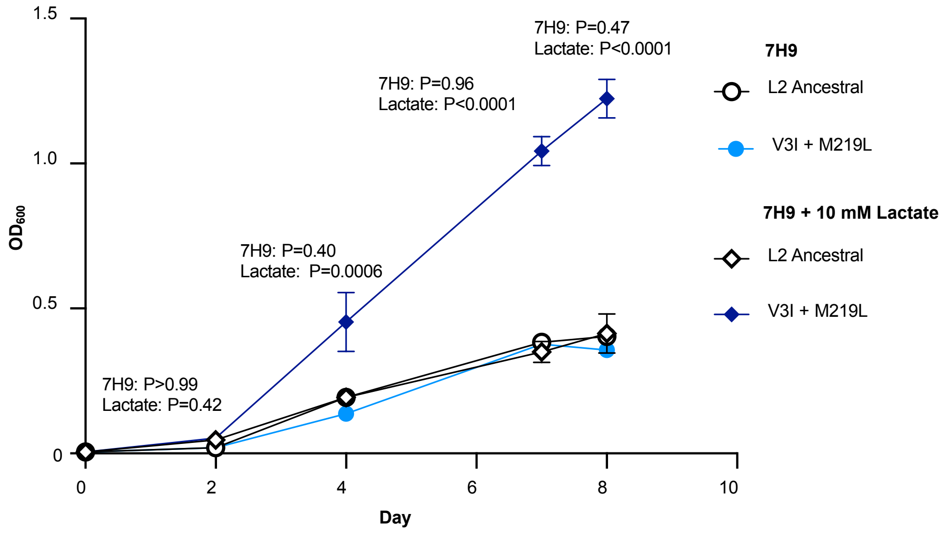

Supplementary Figure 1. Growth curves of L2 Mtb clinical isolates with the indicated *lldD2* alleles. Each dot denotes the average of three technical replicates, the bars represent the standard deviation. The p-value indicates the results of ordinary-one way ANOVA tests conducted at each time point for each media condition, comparing the OD_{600} of the ancestral and variant strains. Sidak's multiple comparison correction was performed.
