# Supplementary material for: Ongoing evolution of the Mycobacterium tuberculosis lactate dehydrogenase reveals the pleiotropic effects of bacterial adaption to host pressure
Source: PLoS Pathog. 2024 Feb 29;20(2):e1012050. doi: 10.1371/journal.ppat.1012050 (PMC10931510; doi:10.1371/journal.ppat.1012050)
Supplement: S2 Fig — Comparison of the area under the curve for the growth curves shown in Fig 3D (A), Fig 3F (B), and Fig 4D (C). Three replicates are shown, error bars indicate the standard deviation. P-values indicate the results of an ordinary one-way ANOVA with Dunnett’s multiple comparison test. Representative of two independent experiments. (PDF) [file ppat.1012050.s002.pdf]

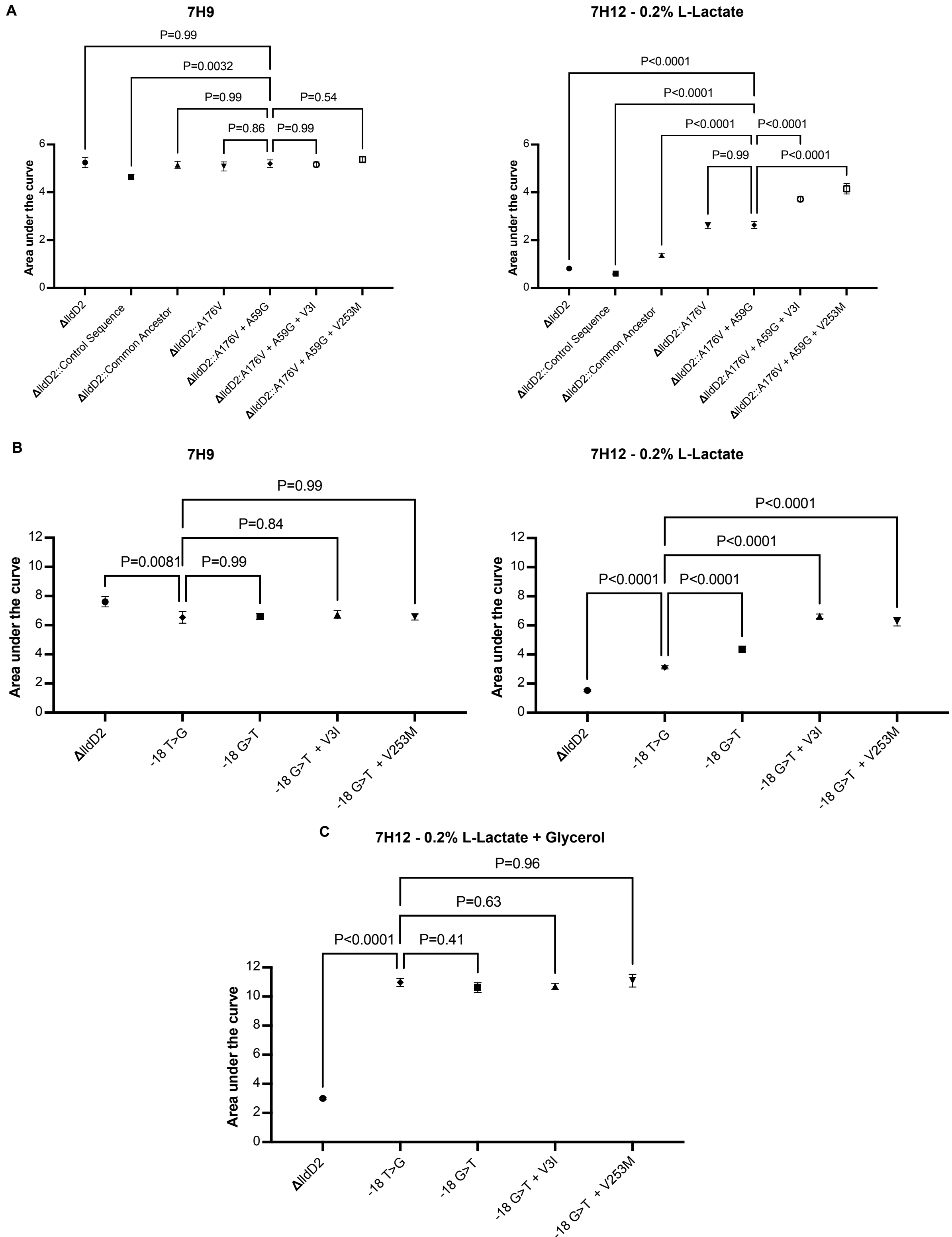

Supplementary Figure 2. Comparison of the area under the curve for the growth curves shown in Fig 3D (A), Fig 3F (B), and Fig 4D (C). Three replicates are shown, error bars indicate the standard deviation. P-values indicate the results of an ordinary one-way ANOVA with Dunnett's multiple comparison test. Representative of two independent experiments.
