# Supplementary material for: Ongoing evolution of the Mycobacterium tuberculosis lactate dehydrogenase reveals the pleiotropic effects of bacterial adaption to host pressure
Source: PLoS Pathog. 2024 Feb 29;20(2):e1012050. doi: 10.1371/journal.ppat.1012050 (PMC10931510; doi:10.1371/journal.ppat.1012050)
Supplement: S3 Fig — P-value indicates the results of two-way ANOVA with Dunnett’s multiple test correction. Triplicate replicates shown, error bars represent the standard deviation. (PDF) [file ppat.1012050.s003.pdf]

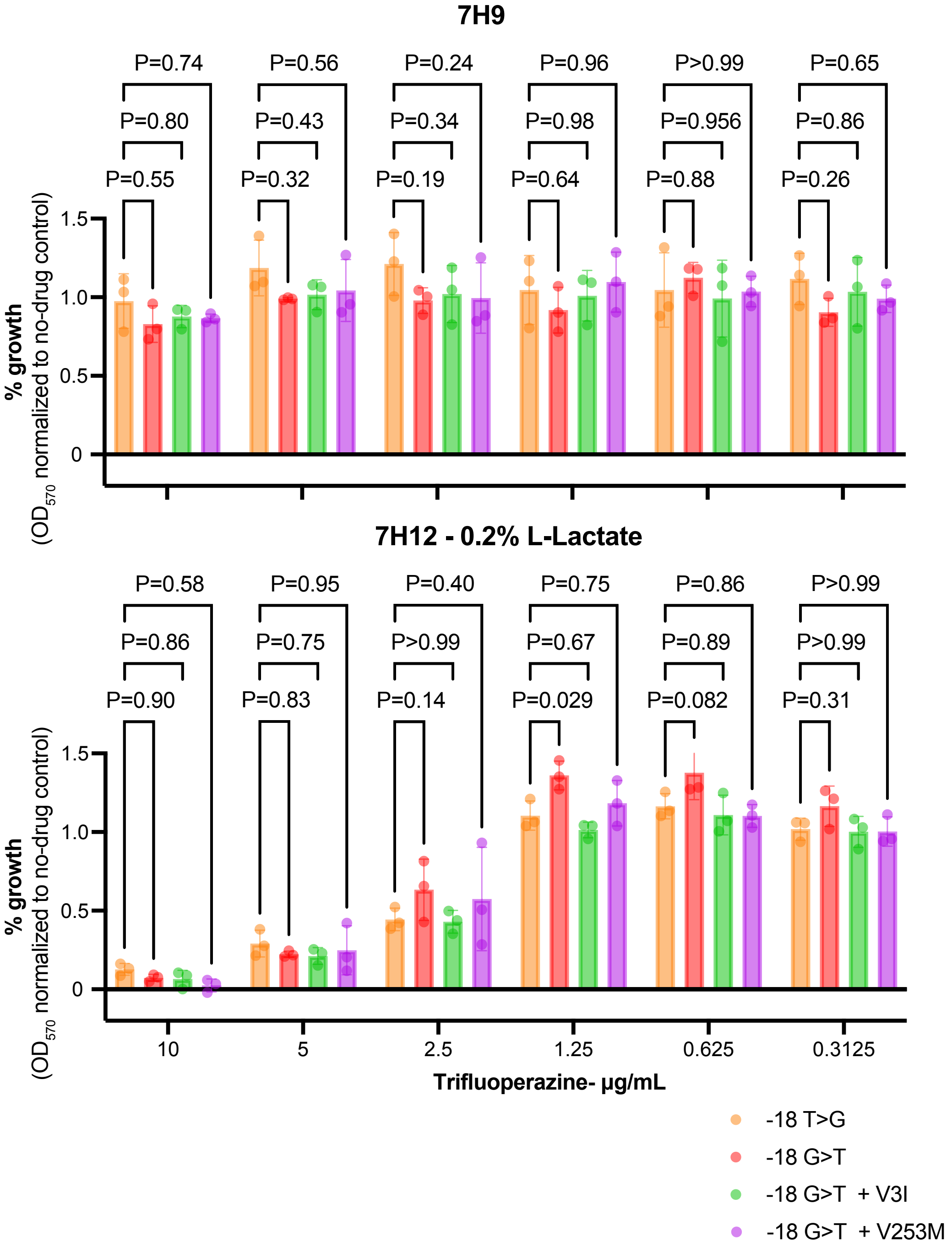

Supplementary Figure 3. Alamar blue assay of trifluoperazine. P-value indicates the results of two-way ANOVA with Dunnett's multiple test correction. Triplicate replicates shown, error bars represent the standard deviation.
