# Supplementary material for: Ongoing evolution of the Mycobacterium tuberculosis lactate dehydrogenase reveals the pleiotropic effects of bacterial adaption to host pressure
Source: PLoS Pathog. 2024 Feb 29;20(2):e1012050. doi: 10.1371/journal.ppat.1012050 (PMC10931510; doi:10.1371/journal.ppat.1012050)
Supplement: S4 Fig — (A) Growth curves of the lldD2 allelic variants with glycerol as the sole carbon source. All cultures started at OD600 0.005 at day 0. Triplicate replicates shown, error bars represent the standard deviation. Representative of two independent experiments. (B) Area under the curve analysis of the growth curves shown in (A). Three replicates are shown, error bars indicate the standard deviation. P-values indicate the results of an ordinary one-way ANOVA with Dunnett’s multiple comparison test. (PDF) [file ppat.1012050.s004.pdf]

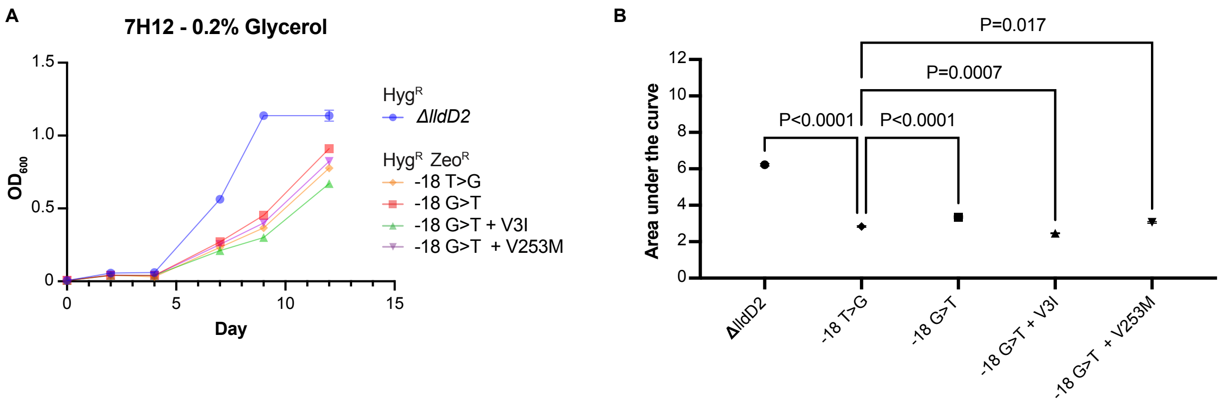

Supplementary Figure 4. (A) Growth curves of the *lldD2* allelic variants with glycerol as the sole carbon source. All cultures started at OD_{600} 0.005 at day 0. Triplicate replicates shown, error bars represent the standard deviation. Representative of two independent experiments. (B) Area under the curve analysis of the growth curves shown in (A). Three replicates are shown, error bars indicate the standard deviation. P-values indicate the results of an ordinary one-way ANOVA with Dunnett's multiple comparison test.
