# Supplementary material for: Ongoing evolution of the Mycobacterium tuberculosis lactate dehydrogenase reveals the pleiotropic effects of bacterial adaption to host pressure
Source: PLoS Pathog. 2024 Feb 29;20(2):e1012050. doi: 10.1371/journal.ppat.1012050 (PMC10931510; doi:10.1371/journal.ppat.1012050)
Supplement: S5 Fig — All cultures started at OD600 0.005 at day 0. Triplicate replicates shown, error bars represent the standard deviation. H37Rv carries the -18 G>T lldD2 mutation. KanR refers to kanamycin resistance. P-values indicate the results of unpaired t-tests. Representative of two independent experiments. (PDF) [file ppat.1012050.s005.pdf]

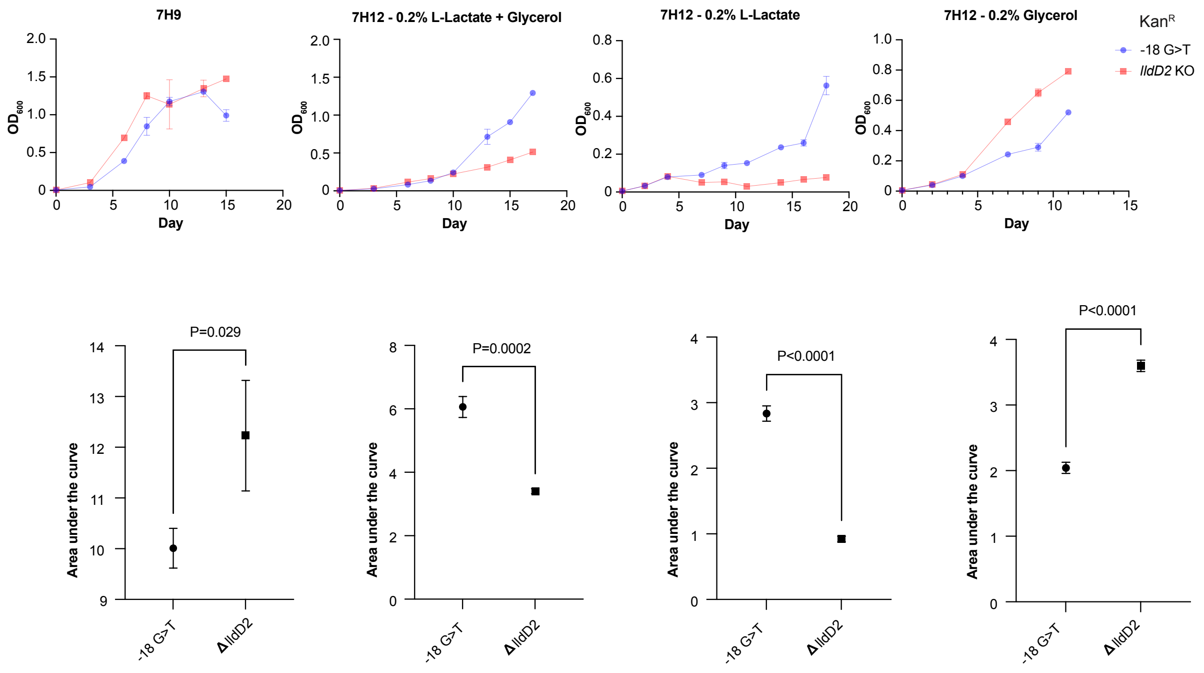

Supplementary Figure 5. Growth curves and corresponding area under the curve analysis for the strains utilized for the metabolic flux assay shown in Fig 4E. All cultures started at OD_{600} 0.005 at day 0. Triplicate replicates shown, error bars represent the standard deviation. H37Rv carries the -18 G>T *lldD2* mutation. Kan^R refers to kanamycin resistance. P-values indicate the results of unpaired t-tests. Representative of two independent experiments.
