# Supplementary material for: Ongoing evolution of the Mycobacterium tuberculosis lactate dehydrogenase reveals the pleiotropic effects of bacterial adaption to host pressure
Source: PLoS Pathog. 2024 Feb 29;20(2):e1012050. doi: 10.1371/journal.ppat.1012050 (PMC10931510; doi:10.1371/journal.ppat.1012050)
Supplement: S6 Fig — The error bars represent the standard deviation of three technical replicates. Blue indicates labeled carbon ions, grey represents total carbon ions. (PDF) [file ppat.1012050.s006.pdf]

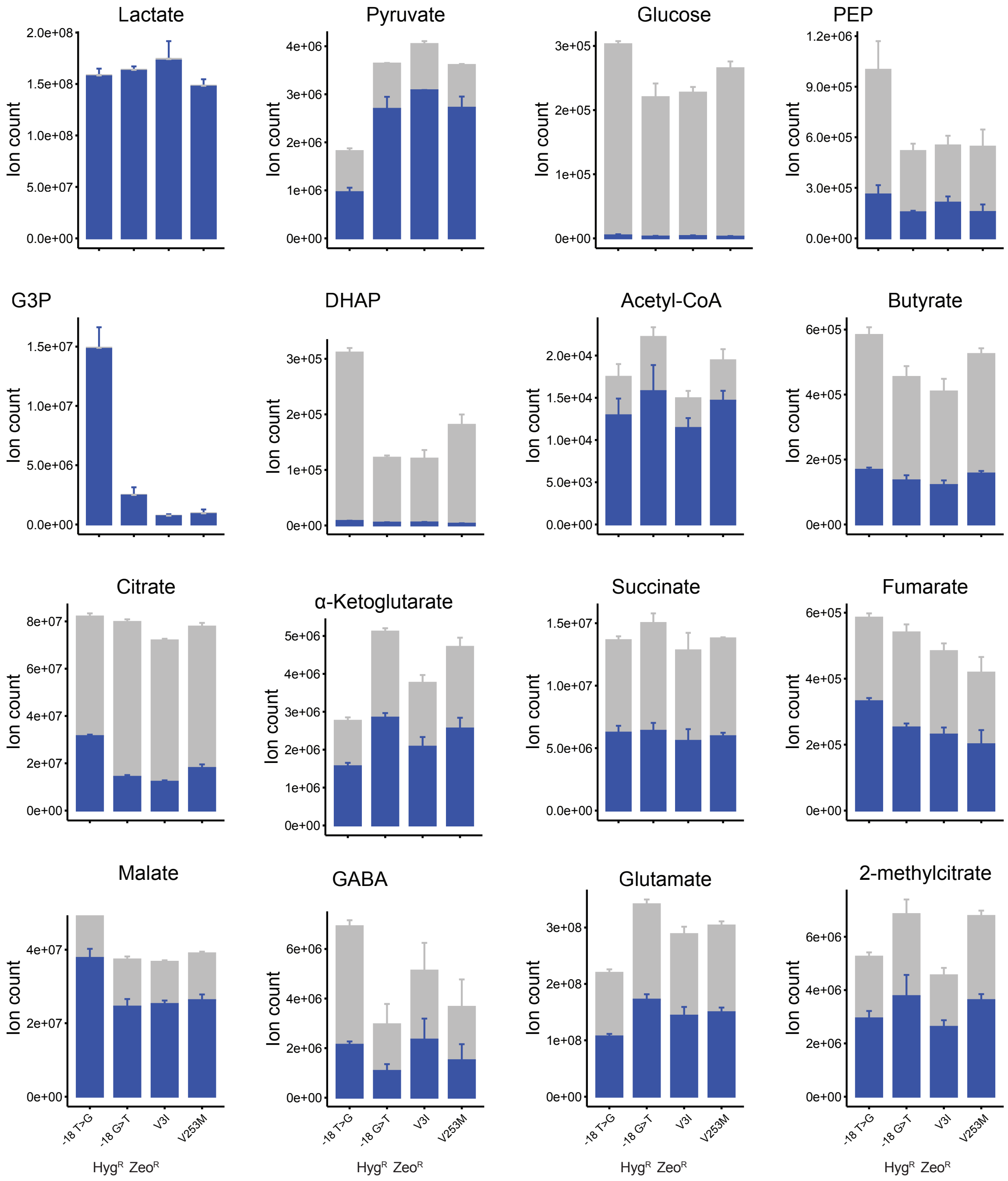

Supplementary Figure 6. ^{13}C-lactate metabolic flux analysis results. The error bars represent the standard deviation of three technical replicates. Blue indicates labeled carbon ions, grey represents total carbon ions.
