# Supplementary material for: Ongoing evolution of the Mycobacterium tuberculosis lactate dehydrogenase reveals the pleiotropic effects of bacterial adaption to host pressure
Source: PLoS Pathog. 2024 Feb 29;20(2):e1012050. doi: 10.1371/journal.ppat.1012050 (PMC10931510; doi:10.1371/journal.ppat.1012050)
Supplement: Attachment — Submitted filename: Response to Reviewers.docx [file ppat.1012050.s011.docx]

**Response to Reviewers**

We thank the reviewers for the thorough assessment of our manuscript and the insightful feedback. We have performed additional experiments and analyses in order to address these comments, which strengthen the overall manuscript. Below we included responses to each point by the reviewers in blue.

**Part I - Summary**
Please use this section to discuss strengths/weaknesses of study, novelty/significance, general execution and scholarship.

Reviewer #1: The work is largely well performed, analyzed and interpreted and delivers novel insights into the evolution of metabolic adaptation of Mycobacterium tuberculosis. The work focuses on lactate metabolism and the lldD2 gene, which encodes a quinone-dependent lactate dehydrogenase. To me, the greatest contribution of this manuscript is the discovery of another enzyme or pathway to catabolize lactate. This might well be another lactate dehydrogenase, that has not been yet characterized, yet, it is novel, and provides some evidence of redundancy, which should agree with the overall hypothesis put forth by the authors, that lactate is an important carbon source for M. tuberculosis.

Strengths:

Carefully done phylogenetic and evolutionary analysis implicating LldD2 and lactate metabolism, as point of evolution in modern mycobacterial strains.
Strong data on genetic manipulation of mycobacteria, demonstrating that lldD2 polymorphisms are indeed affecting lactate metabolism.
RNA-seq analysis reveals genetic programs that have already been implicated in lactate metabolism, directly or indirectly, and therefore provides further evidence of the broad cellular effects imparted by a metabolic defect in lactate metabolism.
 
Weaknesses.

Clofazimine MIC determination should have been carried out in medium without glycerol. And a 2-fold change in MIC is not proof of change (that is noise). The minimum acceptable change in such an assay would be 4-fold. Therefore, I would remove this data from the paper, as it is "negative". Not really supporting the hypothesis, which is otherwise sound.

*and from below:*

*Reviewer #1: The authors need to re-do their CLF MIC determination in medium without glycerol, having lactate as sole carbon source. Changes in growth rate in the mutants should not be a problem.*

We appreciate these questions from Reviewer 1. In these assays, we were using CFZ as a reporter of the quinone reducing activity of LldD2. While we disagree with the reviewer’s assertion that a MIC shift of less than 4-fold is the minimum acceptable change for a MIC assay (our lab and others have shown that MIC changes well below a 4-fold change are a robust phenotype that are associated with clinically meaningful outcomes (Hicks et al. PlosPath 2020; Colangeli et al., NEJM, 2018). In addition, 4-fold MIC shift as a cutoff for susceptibility change may not be sensitive enough to interpret phenotypes conferred by SNPs (Hicks et al. PlosPath 2020; Colangeli et al., NEJM, 2018).

To address concerns regarding the robustness and reproducibility of our results, we note that the original experiment (with lactate and glycerol as carbon sources where response was measured by Alamar blue assessed visually in photographs of each plate) was performed 3 independent times, and each round included 4 different isogenic strains tested in technical duplicates and we observed the same 2-fold shift each time comparing the 3 strains carrying homoplastic variants to the L4 ancestral allele.

We agree with the reviewer that the more informative study is to measure sensitivity to CFZ and trifluoperazine in 7H12 media with lactate as the sole carbon source. Given the reviewer’s concerns about robustness, we here measured responses for each strain at each concentration of drug quantitatively across replicate. We then quantitatively assess the relative growth of the strains with each derived variant to the ancestral allele which allows us to draw conclusions with an expression of statistical confidence. Similar to our previous findings, we find that in media with lactate as the sole carbon source, strains with the derived *lldD2* variants are more sensitive to CFZ but not trifluoperazine. In media with lactate as the sole carbon source, Mtb strains expressing homoplastic *lldD2* variants were significantly more sensitive to CFZ compared to the -18 T>G strain expressing the L4 ancestral allele at concentrations of CFZ ranging from 0.0156-0.0625 μg/ml; the strains with two derived alleles showed greater sensitivity than the strain with the -18 G>T mutation alone. These data support our hypothesis that the derived variants are gain of function. CFZ susceptibility is increased when more electrons flood into the quinone pool where LldD2 is the terminal electron donor. These data are shown below and are now included as the new Figure 4C, main text lines 196-211 and 453-467, and supplementary figure fig. S2.

Physiology and metabolism results are largely good and definitive. I just did not understand why the authors jumped on "methylglyoxal" as the explanation for the phenotypes observed. The authors presented no proof that methylglyoxal is formed or that accumulates. This is similar to what was conveyed in the Pethe study, without any proof. The authors must remove any mention of methylglyoxal of the manuscript. Or show that it is formed and accumulates.

We demonstrated that *ΔlldD2* strains are impaired in growth in lactate, even in the presence of glycerol, which we show is a viable carbon source for these strains. We sought to provide a plausible biologic model for this growth inhibition. In our C13-lactate labeling study, the mutant strains accumulate DHAP and G3P. Previous literature shows build-up of intrabacterial triose is toxic for growth (<https://doi.org/10.1128/MMBR.00123-21>). It has been hypothesized that methylglyoxal contributes to the triose toxicity across the kingdoms of life (<https://doi.org/10.1021/acs.chemrestox.2c00160> and <https://doi.org/10.1042/bst0311406>), including in Mtb (Pethe et el 2010 and <https://doi.org/10.1128/mbio.00663-19>). In the Results section, we simply cite the literature regarding triose toxicity.

However, in the context of the discussion, we do not feel that it is too speculative to discuss our findings in the context of the methylglyoxal model presented in the Pethe et al. paper. Methylglyoxal is a by-product of these sugar phosphates, a well-established process that has been described. It seems to us that it would be poor scholarship not to discuss the Pethe model but agree that this is better framed as a discussion point rather than introduced in the Results section.

Reviewer #2: This manuscript argues that the gene lldD2 (Rv1872c) which encodes a lactate dehydrogenase, has been positively selected for in the human host. Clearly, Mtb has continued to evolve, with selective pressures, but the authors claim that BCG vaccination may have played a role in acquiring these mutations, but no animal experiments have been included to test their hypothesis. As an alternative to host immune selective pressure, the authors have not described that lactate dehydrogenase converts pyruvate to lactate with the concomitant conversion of NADH to NAD+. Since resistance to Isoniazid and Ethionamide can be mediated by alterations in the NADH/NAD ratios, could not these drugs play a role in the evolution of the mutations in lldD2? These possibilities are not considered.

We are grateful for the thorough responses shared by Reviewer 2.

We think the first part of this question may reflect a misunderstanding about the biochemical activity of LldD2. Our data is consistent with other literature that indicates that LldD2 utilizes quinone rather than NAD+ as cofactor. LldD2 is a member of the well-described and closely-related *Corynebacterium glutamicum* quinone-dependent LDH (<https://doi.org/10.1128/jb.01147-07>); it is not a NADH-dependent lactate dehydrogenase. LldD2 has an annotated FMN and 2-oxocarboxylate binding site, suggesting that FMN shuttles electrons from lactate to a quinone; NADH is not involved. We have edited our language in the main text at lines 64-65 to make this clearer.

In addition, *lldD2* selection occurred at the root positions of MTBC phylogeny, which predates the utilization of isoniazid and ethionamide by thousands of years, so it is unlikely that these antibiotics are the primary drivers of *lldD2* diversification. Further, our group completed a GWAS to identify Mtb variants associated with INH and ETH resistance and no link to *lldD2* was identified (<https://doi.org/10.1038/s41564-018-0218-3> and <https://doi.org/10.1128/mbio.00616-19>).

However, this question led us to a secondary question -- could there be potential secondary changes to the NAD+/NADH ratio that might alter INH or ETH sensitivity and that variants driven by non-drug selection could have altered drug sensitivities. Therefore, we completed alamar blue MIC assays in 7H12 – 0.2% lactate and 7H12 – 0.2% lactate + glycerol and saw no difference in susceptibility between the L4 ancestral -18 T >G variant and the homoplastic variants. Because these experiments were so speculative, not well connected to LldD2 activity, and would be best repeated under a variety of conditions, we did not include these data in the paper.

Also, the use of 7H12 for some of the assays is not explained and this could be playing a role in the mutations examined in their analyses. Further clarifications will be needed.

*and from below*

*Is this because the strains fail to grow on 7H9 with lactate? 7H12 has casein and I would hypothesize that lldD2 mutants could grow on 7H12 with lactate, but not on 7H9 with lactate because of the alteration of the NADH/NAD+ ratios in the mutants.*

*Could the alterations in lactate dehydrogenase activities be causing the strains to acquire auxotrophic phenotypes?*

We have combined our response to the 7H12 question and the auxotrophy question because we feel that they scientifically fit together – and that the auxotrophy question is very interesting. The differences between 7H9 and 7H12 media are the presence of casamino acids in 7H12 vs 7H9 media which is supplemented with oleic acid, dextrose, albumin, and catalase. 7H9 also includes with 0.2% glycerol while we used 0.2% lactate +/- glycerol as indicated. When lactate is present, the gain-of-function phenotype of the evolved *lldD2* variants phenotype is observed in both 7H9 and 7H12. As shown below, we used 7H9 supplemented with 10 mM lactate and completed growth curves with clinical strains. We find that strains carrying the L2 ancestral *lldD2* allele or homoplastic variants demonstrate similar growth rates in standard 7H9, but the strain expressing homoplastic mutations grows faster in the presence of lactate. These data are now referenced in the main text at lines 144-155 and included in supplementary figure fig. S1.

We have used 7H12 as our base media for single carbon source studies both in this work and in previously published studies (Hicks et al -prpR). The simple answer is that 7H12 allows us to more precisely attribute the phenotypes of *lldD2* variants to lactate. This was clarified at lines 151-152.

The more complex question is why the presence of casamino acids allows growth of all Mtb strains in single carbon source media. While we consider this question outside the scope of this manuscript, we do think that this could be due to a carbon-source dependent autotrophy and thank the Reviewer for this idea which we will explore in future work.

Reviewer #3: The manuscript presents a detailed analysis of genetic variation of the lldD2 gene in Mycoabcterium tuberculosis. First by assessing natural variation in clinical strains and then by introducing the variants by allelic replacement and assessing the phenotype. In general the manuscript is very well written. To the best of my knowledge in silico and in vitro analysis are nicely done and show a very nice example of the continuous adaptation of the bacteria to host challenges.

We thank Reviewer 3 for the feedback.

**Part II – Major Issues: Key Experiments Required for Acceptance**
Please use this section to detail the key new experiments or modifications of existing experiments that should be absolutely required to validate study conclusions.

Generally, there should be no more than 3 such required experiments or major modifications for a "Major Revision" recommendation. If more than 3 experiments are necessary to validate the study conclusions, then you are encouraged to recommend "Reject".

Reviewer #1: The authors need to re-do their CLF MIC determination in medium without glycerol, having lactate as sole carbon source. Changes in growth rate in the mutants should not be a problem.

As described above, we agree and we completed this. Similar to our previous findings, we find that in media with lactate as the sole carbon source, strains with the derived *lldD2* variants are more sensitive to CFZ but not trifluoperazine. These data support our hypothesis that the derived variants are gain of function (CFZ susceptibility is increased when more electrons floods into the quinone pool where LldD2 is the terminal electron donor). Please see new Figure 4C, main text lines 196-211 and 453-467, and supplementary figure fig. S2.

The authors must remove any mention that methyglyoxal is toxic to Mtb and differentially toxic under their experimental conditions as no direct proof is provided of increase methylglyoxal formation or accumulation.

As we wrote above:

We demonstrated that *ΔlldD2* strains are impaired in growth in lactate, even in the presence of glycerol, which we show is a viable carbon source for these strains. We sought to provide a plausible biologic model for this growth inhibition. In our C13-lactate labeling study, the mutant strains accumulate DHAP and G3P. Previous literature shows build-up of intrabacterial triose is toxic for growth (<https://doi.org/10.1128/MMBR.00123-21>). It has been hypothesized that methylglyoxal contributes to the triose toxicity across the kingdoms of life (<https://doi.org/10.1021/acs.chemrestox.2c00160> and <https://doi.org/10.1042/bst0311406>), including in Mtb (Pethe et el 2010 and <https://doi.org/10.1128/mbio.00663-19>). In the Results section, we simply cite the literature regarding triose toxicity.

However, in the context of the discussion, we do not feel that it is too speculative to discuss our findings in the context of the methylglyoxal model presented in the Pethe et al. paper. Methylglyoxal is a by-product of these sugar phosphates, a well-established process that has been described. It seems to us that it would be poor scholarship not to discuss the Pethe model but agree that this is better framed as a discussion point rather than introduced in the Results section.

Reviewer #2: Specific questions to the authors:
1. In the list of 296 strains, were any of them drug resistant?

These strains were obtained from population-based surveillance studies of drug resistance, so some of these strains are indeed drug resistant (<https://doi.org/10.1038/s41559-018-0680-6>). We clarify this in the main text lines 105-108.

2. You make a point that 99.6% of the 59,00 strains analyzed have mutations in lldD2 gene compared to the 10% in the lldD1 gene. Are there no other genes of the 4000 genes common to the 50,000 strains that possess this amount of divergence? Is this only found in lldD2?

We compared the prevalence of *lldD2* mutations to *lldD1* because both are members of the FMN-dependent alpha-hydroxy acid dehydrogenase family (main text lines 130-137). We did not intend to suggest that *lldD2* is the only gene demonstrating fixation of ancestral root mutations and homoplastic variants. While we have not analyzed the frequency of every variant across the 4,000 genes common to the 50,000 strains, a very interesting endeavor for future studies, the large prevalence of mutations in *lldD2* driven by a combination of ancestral + contemporary mutations. Root mutations in successful sub-lineages explain much of the high prevalence of *lldD2* variants. Thus, *lldD2* is not representative of genes undergoing strong positive selection that only began recently. For example, canonical drug resistance genes show a large signal for recent positive selection compared to *lldD2* (<https://doi.org/10.1126/science.abq2787>), but only a minority of Mtb strains carry variants in these genes. We have edited the main text at lines 134-137 to clarify our intent given the Reviewer’s questions #1-3.

3. For the experiments with the 10 clinical isolates with lldD2 mutations, what are the genotypes for other drug resistances?

These clinical isolates are phenotypically sensitive to INH, RIF, SM, and EMB. In addition, we did not identify genetic variants known to mediate resistance to these drugs. These strains are now noted to be drug sensitive at lines 139-141.

4. You note that the strains are grown on 7H12 with lactate but give no explanation as to why you switched to 7H12. Is this because the strains fail to grow on 7H9 with lactate? 7H12 has casein and I would hypothesize that lldD2 mutants could grow on 7H12 with lactate, but not on 7H9 with lactate because of the alteration of the NADH/NAD+ ratios in the mutants. Such a phenotype has been observed in M. smegmatis mutants that were selected to be resistant to Isoniazid and ethionamide when selected on Mueller Hinton agar. The mutantations map to ndh and the strains have altered NADH/NAD ratios which caused serine/glycine auxotrophies by inactivating the first step of serine biosynthesis (Miesel, L et al. “NADH dehydrogenase defects confer isoniazid resistance and conditional lethality in Mycobacterium smegmatis.” Journal of bacteriology vol. 180,9 (1998): 2459-67. doi:10.1128/JB.180.9.2459-2467.1998). Could the alterations in lactate dehydrogenase activities be causing the strains to acquire auxotrophic phenotypes?

As mentioned above, the strains all grow on 7H9 (OADC) with lactate. We used 7H9 supplemented with 10 mM lactate and completed growth curves with clinical strains. Similar to what is shown in Fig. 3A, we find that strains carrying the L2 ancestral *lldD2* allele or homoplastic variants demonstrate similar growth rates in standard 7H9, but the strain expressing homoplastic mutations grows faster in the presence of lactate.

As described above, we used 7H12 because we wanted to utilize a single carbon source media which allowed us to evaluate the effect of lactate more precisely vs 7H9 which has a more complex mixture of carbon sources by virtue of OADC supplementation. We clarify this at lines 151-152 of the main text.

5. It would have been best to make the mutants in the isogenic strains in the clinical isolates from Vietnam. Also, transformants were made using an L5 integration vector with a kanamycin-resistance gene in a vector that has yet to be published. Is this a derivative from the Lee et al paper or the Stover et al, paper and was the integrase gene still in the vector. What was the value of the bar codes/ Were the transformants selected on 7H12? If selected on 7H9, did you select for lldD2 reversions?

We appreciate the suggestion regarding the use of clinical strains. It is unclear if the reviewer is referring to the transformations of the clinical strains or the recombinant *lldD2* variants. For the former, the plasmid is a derivative of the pJEB402 plasmid originally described by Lee et al (<https://doi.org/10.1128/mbio.00312-17> and <https://doi.org/10.1128/jb.00137-18>); this construct is described by Martin et al. This is cited in the methods section of the main text, see lines 395-400. The clinical strains we used were originally barcoded for use in a separate study (<https://doi.org/10.1101/2023.04.09.536166>). All transformants were selected on 7H10 (solid media 7H9) and the integrase gene remained in the vector. This is now described in the methods at lines 390-392. For the recombinant *lldD2* variants, we did not select for *lldD2* reversions; it would be interesting to identify potential genetic interactions in future studies.

6. The problem with the H37Rv constructs are the notorious problem of PDIM loss when culturing H37Rv in Middlebrook 7H9. The Berney lab has a recent paper, but unless you genome sequenced the strains after even a simple transformation, you could be selecting for PDIM. I suspect that the RNAseq data showing overexpression of PDIM genes is not due to lldD2h mutations but rather the acquisition of PFIM mutations. Unfortunately, this makes it impossible to know if the differences in transcription result from lldD2 mutations alone.

We whole-genome sequenced the strains after the transformations to identify second site mutations including PDIM gene mutations. Comparing the -18 T>G and -18 G>T strains used in the RNAseq, the only second site mutation identified is a V624A mutation in *pks12*, which is not implicated in PDIM synthesis (https://doi.org/10.1084/jem.20041429). The mutation is found in the -18 G>T strain, which phenocopies -18 G>T + V3I and -18 G>T + V253M (along with the step-wise variants), so it is unlikely that this mutation is driving the PDIM gene expression signature.

7. While clofazimine has been suggested to alter electron flow, the Nuo complex is not essential for the growth of Mtb in vitro or in mice. This makes the clofazimine argument challenging to interpret.

As mentioned previously, LldD2 is annotated as quinone-dependent LDH. Therefore, we did not complete the assay because CFZ is implicated in electron flow, but instead to probe the quinone activity of the LldD2 variants given that CFZ functions as a substrate for NDH-2, a menaquinone oxidoreductase (<https://doi.org/10.1074/jbc.M110.200501>) like LldD2. We observe the same MIC to CFZ in standard 7H9, but find that compared to an ancestral variant, the homoplastic variants are more susceptible in 7H12 - lactate + glycerol or with lactate as the sole carbon source. As a control we also assessed trifluoperazine, which also targets NDH-2 but not as quinone competitor (https://doi.org/10.1073/pnas.0500469102); accordingly the ancestral and homoplastic variants do not demonstrate a lactate-dependent difference in susceptibility. This suggests that the variants may impact the quinone activity of LldD2. See main text lines 196-211.

8. The possibility of the in vivo selection for lldD2 mutations in humans is very intriguing. However, just growth enhancement in vitro is not sufficient evidence for this hypothesis to be justified. Do you have any evidence that lldD2 mutations cause a survival advantage that prevents killing? Most all drug resistance mutations provide a resistance to a killing assault.

The evolutionary analysis of clinical strain genomes demonstrates selection in human infection – this is not a hypothesis. The two main selective pressures faced by Mtb are host challenge and antibiotic stress. *lldD2* has been undergoing positive selection thousands of years before the introduction of antibiotic stress, so host environment must be the driver of the diversification. The *in vitro* experiments are not designed to justify host selection, instead they provide a model to explain why *lldD2* variants are advantageous to Mtb in host-relevant environmental conditions. This logic is referenced in main text lines 84-89.

9. The argument that lldD2 is modified significantly more than the other 4000 genes of Mtb would be an interesting paper, but has this not been argued in the cited papers?

We did not imply that we were the first to suggest that *lldD2* is modified significantly more than other genes and we were sure to cite and highlight the contributions of previous work, see lines 95-103. Our study adds novelty by illustrating the stepwise nature of *lldD2* evolution and demonstrating the prevalence across all lineages.

Reviewer #3: Regarding natural variation. The narrative in the introduction suggests that what we are looking to a process of positive selection. While this is likely true for many variants, and particularly homoplastic or other recently arisen, some others, particularly at the root of the tree may have fixed by other processes related to drift (bottlenecks, founder effects….etc). Another possibility is that some changes are compensatory to others (early changes are now detrimental so strains are trying to compensate the fitness cost). Somewhat this is discuss later including the fact that probably the pathogen is facing a dynamic fitness landscape what leads to my second comment

The suggestion is that adaptation is probably linked to lactate accumulation in the macrophages. Is there any information about how lactate levels may change during infection or even in healthy conditions? Are also reported differences between human genetic backgrounds that may explain the early fixation of mutations you see in the phylogeny?

Finally, in terms of analysis, the suggestion is that lldD2 mutations may help the bacteria to better survive host stresses which in turn may help the bacteria to spread between host. While transmission is a complex phenotype I am wondering if there is some hint in your dataset (probably from Vietnam?) that strains carrying the mutation are more likely to be in cluster (or have shorted branch length as a surrogate)

We agree with the reviewer’s suggestion that drift and compensatory mutations are important alternative models, so we included these possibilities in the discussion (lines 295-302). Indeed, we created the panel of stepwise *lldD2* variants to test this very question: are *lldD2* mutations functional and have an additive effect, or are they products of genetic drift/compensatory evolution?

In the conclusion, we now suggest that future work should address the question you raised concerning the impact of potential differences in human genetic backgrounds in genes that regulate lactate metabolism (<https://doi.org/10.1038/s41392-022-01151-3>, <https://doi.org/10.1016/j.redox.2020.101454>, and <https://doi.org/10.1038/s41467-019-09458-6>) and how this may contribute to the early fixation of *lldD2* variants (main text lines 342-346).

We appreciate the Reviewer’s question about where lactate is relevant in the course of infection. In the introduction, we note that lactate accumulates in macrophages during infection upon IFN-γ stimulation (doi: 10.4049/jimmunol.1600266) (lines 76-78). In terms of healthy conditions, lactate is a circulating nutrient that is a major contributor to the TCA cycle in several organs, but especially the lung (https://doi.org/10.1016/j.cmet.2020.07.013 ) (lines 81-82). A previous study has already demonstrated that the *lldD2* promoter mutation is associated with increased transmission of L4 strains, this is referenced in the main text at lines 64-65 (https://doi.org/10.1126/sciadv.aat5869). In this work, they compared number of descendants per node.

**Part III – Minor Issues: Editorial and Data Presentation Modifications**
Please use this section for editorial suggestions as well as relatively minor modifications of existing data that would enhance clarity.

Reviewer #1: No minor issues.

Reviewer #2: More details on the genetic manipulations should be included. It is unclear how the mutations in the lldD2 were selected for after recombineering.

Our method followed the RecET recombination protocol published previously and this was cited in the methods (<https://doi.org/10.1007/978-1-4939-2450-9_10>). However, we added additional detail, please refer to the methods lines 357-362.

Reviewer #3: (No Response)
